# Childhood Influenza Vaccination Is Not a Priority for Parents: A National, Cross-Sectional Survey of Barriers to Childhood Influenza Vaccination in Australia

**DOI:** 10.3390/vaccines13050540

**Published:** 2025-05-19

**Authors:** Maryke S. Steffens, Jessica Kaufman, Katarzyna T. Bolsewicz, Suzanna Vidmar, Maria Christou-Ergos, Majdi M. Sabahelzain, Julie Leask, Justin Boxall, Frank Beard, Margie Danchin

**Affiliations:** 1 National Centre for Immunisation Research and Surveillance, Kids Research, Sydney Children’s Hospitals Network, Westmead, NSW 2145, Australia; 2 The Children’s Hospital at Westmead Clinical School, Faculty of Medicine and Health, The University of Sydney, Sydney, NSW 2145, Australia; 3 Vaccine Clinical Trials and Uptake Group, Murdoch Children’s Research Institute, Royal Children’s Hospital, Parkville, VIC 3052, Australia; 4 Department of Paediatrics, Royal Children’s Hospital, The University of Melbourne, Parkville, VIC 3052, Australia; 5 Sydney School of Public Health, Faculty of Medicine and Health, The University of Sydney, Sydney, NSW 2006, Australia; 6 Clinical Epidemiology and Biostatistics Unit, Murdoch Children’s Research Institute, Royal Children’s Hospital, Parkville, VIC 3052, Australia; 7 Sydney Infectious Diseases Institute, Westmead Hospital, Westmead, NSW 2145, Australia; 8 Department of General Medicine, Royal Children’s Hospital, Parkville, VIC 3052, Australia

**Keywords:** influenza vaccine, vaccination, access and acceptance barriers, national survey, Australia

## Abstract

**Background/objectives**: Influenza vaccines are recommended and free in Australia for children aged <5 years, but uptake remains low at 25.8% compared to the targets of 40% and 50%. National data on barriers hindering paediatric influenza vaccination can inform strategies to improve uptake. The aim of this study was to measure barriers to influenza vaccination in Australian children aged <5 years. **Methods**: A national, cross-sectional survey of parents of children aged <5 years was conducted in March/April 2024. Parents were recruited using an online panel and asked about their intention to get an influenza vaccine for their youngest child in the upcoming influenza season. An adapted version of the validated Vaccine Barriers Assessment Tool measured 14 influenza vaccination barriers. Analysis assessed the prevalence of barriers and differences between parents intending to and those unsure or not intending to vaccinate by calculating the prevalence difference and 95% confidence interval. **Results**: A total of 2000 parents were recruited nationally. The most common barrier was parents feeling distressed when thinking about vaccinating their child against influenza (66.1% of intending parents, 65.6% of unsure/not intending parents). The barrier with the largest difference between intending and not intending/unsure parents was not prioritising their child’s influenza vaccination (47.2% vs. 6.1%, PD = 41.1 ppts, 95% CI: 35.9%, 46.3%). Other barriers with large differences were parents not feeling guilty if their unvaccinated child got influenza (41.5% vs. 7.5%, PD = 34.0 ppts, 95% CI: 28.8%, 39.1%) and parents not believing that influenza vaccines are effective (31.3% vs. 3.0%, PD = 28.2 ppts, 95% CI: 23.6%, 32.9%). **Conclusions**: Parents should be encouraged and supported to prioritise influenza vaccination alongside routine childhood vaccines in campaigns that emphasise disease risk and the importance, safety and effectiveness of influenza vaccination, and by optimising access to influenza vaccination. We recommend conducting similar surveys regularly to monitor trends in parental barriers to childhood influenza vaccination.

## 1. Introduction

Influenza vaccination for young children aged 6 months to 5 years has been recommended and funded in Australia under the National Immunisation Program (NIP) since 2020 [1]. While no national targets for influenza vaccination coverage in young children are currently in place in Australia, the target of 40% coverage and the ‘stretch target’ of 50% from the most populous state in Australia, New South Wales [2], can be used as an appropriate proxy. At a national level, however, only 25.8% of this age group were vaccinated in 2024 [3]. Coverage in Australia has improved in recent years: in 2017, uptake was just 5.0% [4], increasing considerably in 2018 and 2019 to 25.9% and 41.8%, respectively [4,5], in part due to the introduction of state and nationally funded programmes [6], and peaking at 45.2% in 2020 during the COVID-19 pandemic [7]. However, vaccination rates have since been on the decline. By comparison, coverage rates in young children for the 2023-24 season in similar high-income countries such as the United States and England were 60.8% (children aged < 5 years) and 44.4% (children aged 2–3 years), respectively, although recent declines have also been reported [8,9,10].

In Australia, the influenza season typically occurs between May and September each year [11]. Young children <5 years are at higher risk of experiencing severe influenza-related disease requiring hospitalisation than all other age groups in Australia other than adults aged ≥65 years, being hospitalised at a rate of 283.5 per 100,000 population in infants aged <1 year and 124.1 per 100,000 population per year in children aged 1–4 years [12]. Children with comorbidities are hospitalised at a higher rate than healthy children, although a substantial proportion of hospitalisations (between 55 and 60%) involve healthy children [13,14,15]. Complications from influenza for young children, especially those with comorbidities, can be severe, requiring admission to intensive care units, and include pneumonia, encephalitis, acute renal failure, myocarditis and death [15].

While vaccination is moderately effective for preventing influenza infection in young children (vaccine effectiveness is approximately 65% in children <5 years when the vaccine and circulating strains are well matched [1]), importantly, vaccination protects against severe disease and hospitalisation [16,17]. For example, the 2024 Southern Hemisphere seasonal influenza vaccine reduced the risk for influenza-associated hospitalisation among high-risk groups (including young children) by 34.5% [18]. Additionally, there is some evidence that vaccination protects the wider community and individuals at risk of severe disease [19]. Given these benefits, the suboptimal and declining uptake in children <5 years is concerning.

Suboptimal uptake of childhood vaccines is influenced by a broad range of factors [20]: parents and caregivers (hereafter “parents”) may face practical barriers to accessing appointments and affording costs, i.e., the cost of travel or seeing a doctor, and may have negative thoughts and feelings or social influences related to vaccination that affect motivation [21]. These factors are evident in research among Australian parents, which has found parents can experience difficulties obtaining influenza vaccination appointments for their children, especially outside metropolitan areas [22]. There is further evidence that some parents experience out-of-pocket costs when seeking influenza vaccination for their children, such as general practice (GP) consultation fees charged when administering the vaccine and lost paid work hours when attending the appointment [23]. Additionally, parents have reported the burden of having to attend influenza vaccination appointments annually when faced with competing priorities such as needing to work or attend to their child’s health needs beyond vaccination [22,24]. Some parents are not aware of the recommendation that all children aged <5 years should receive the influenza vaccine annually [25]. Beyond practical issues, there is evidence that some Australian parents are not aware of the potential severity of influenza in young children and thus are not concerned about their child contracting influenza; others have doubts about the effectiveness of influenza vaccination and concerns about influenza vaccination safety [22,23]. Some parents are less motivated to vaccinate their children against influenza because it is neither a common practice and not expected among their peers, nor recommended routinely by their healthcare provider [22,23].

To address low coverage, it is essential to understand the factors that hinder childhood influenza vaccination, as this knowledge can inform strategies to encourage uptake. To date, in Australia, however, parental barriers have not been systematically or comprehensively measured in a way that reveals which barriers are associated with the intention to vaccinate and vaccination behaviour and are therefore most useful for informing interventions to increase uptake, or in a way that allows tracking over time. Therefore, the aim of this study was to measure the prevalence of parental barriers to influenza vaccination in young children aged <5 years and compare this between parents with different vaccination intentions for the 2024 influenza season. A secondary aim was to compare the prevalence of barriers between parents with different key demographic variables. This study contributes to a broader goal of collecting data on childhood influenza vaccination barriers on an ongoing basis.

## 2. Methods

### 2.1. Study Design

A cross-sectional survey design assessed influenza vaccination barriers in Australian children <5 years of age.

### 2.2. Setting

The setting was a national online survey platform, hosted by a national panel provider (i-Link). We collected data during March–April 2024, just before the start of the influenza season.

### 2.3. Participants

Participants were adults ≥18 years of age, living in Australia, who could read English and who were the parent or carer of a child <5 years. Participants were excluded if their youngest child was ≥5 years or if they could not complete the survey in English.

The sample was derived from an online panel of over 120,000 Australian adults (i-Link), which is constructed to represent the Australian general community. The panel provider recruits members through online and offline means such as print media, online marketing initiatives, direct mail, affiliate partnerships and personal invitations, and is nationally representative in terms of state and territory of residence. The panel maintains detailed information about members to aid study recruitment. For this study, the panel provider randomly selected a sample stratified by state/territory of residence and invited them to participate by sending a notification to their dashboard; invited participants who logged on when the study was open saw the notification. Participation was voluntary. The survey remained open until the sample size was reached. Only one person per household (determined by IP address) could participate. A unique session ID ensured anonymity and one-time survey access.

A total of 2000 participants completed the survey. A reliable denominator required to calculate the response rate was not available because the panel provider was not able to provide accurate information about the proportion of invited participants who logged on and saw the notification. The final sample distribution reflected Australian Bureau of Statistics Census 2021 data by state/territory.

### 2.4. Variables

The primary outcome was parental reports of barriers to influenza vaccination for their youngest child, assessed by their agreement with statements from the Vaccination Barriers Assessment Tool (VBAT). The group variable was parental intention to vaccinate their child against influenza in 2024. Sociodemographic characteristics collected in the survey included parent’s age, gender, location, number and age of children, education, Aboriginal and/or Torres Strait Islander status, language spoken at home, household financial stress and whether their child had previously had an influenza vaccine.

### 2.5. Data Sources and Measurement

The survey was conducted in English. All data were self-reported by participants. Participants responded to 14 VBAT statements about their experience of vaccinating their youngest child against influenza (e.g., ‘It is easy to get an appointment when my child’s flu vaccination is due’) using a 5-point Likert scale. The VBAT, developed and validated in Australia, draws on known drivers of vaccination to predict vaccine uptake [26]. While the VBAT was developed for measuring barriers to routine childhood vaccinations with parents of children <5 years of age, it is an appropriate tool to use in this context because it captures key acceptance- and access-related factors that influence parental vaccination behaviour, which are also relevant to decisions about influenza vaccination. We adapted statements for influenza vaccination by minimally modifying the wording. Parental intention to vaccinate was assessed using an adapted question from the Behavioural and Social Drivers of Vaccination survey tool, using a ‘yes’, ‘no’ or ‘unsure’ scale. Sociodemographic information and prior influenza vaccination were collected using multiple-choice questions. Financial stress was assessed using a question from the Household, Income and Labour Dynamics in Australia (HILDA) survey. Participants selecting two or more financial stress indicators were categorised as experiencing financial stress.

### 2.6. Bias

Selection bias was minimised through random sampling stratified by state/territory and weighting by geographic and demographic factors. Measurement bias was minimised by using validated instruments (VBAT) and standardised questions. There was the potential for recall bias regarding vaccination history.

### 2.7. Study Size

We aimed to recruit a national sample of 2000 participants. This sample size had an 80% or greater power to detect a difference in prevalence of 10% between two groups where the ratio of sample sizes was 1:8, at a two-sided 5% significance level. The margin of error for estimating a single prevalence was within 3 percent.

### 2.8. Quantitative Variables

Quantitative data (age, number of children) were categorised. All remaining data collected were categorical.

### 2.9. Statistical Analysis

We performed analysis using Stata version 18.0 (StatCorp, College Station, TX, USA, 2023). To perform analysis of the barriers to vaccination, we inverted positively framed vaccination statements and responses to a negative framing (‘It is not easy to get an appointment when my child’s flu vaccination is due’) and collapsed the responses into two categories: agree (strongly and slightly agree) and disagree (strongly and slightly disagree) to focus on the presence or absence of barriers and facilitate the interpretation of prevalence differences. We treated the low number of ‘can’t say/don’t know’ respondents as missing data. We collapsed influenza vaccination intention responses into two categories: yes and no/not sure. There was no missing data for intention and sociodemographic variables.

To make the data more representative of the target population, we weighted the data by state/territory, location (urban and rural areas, based on the Australian Statistical Geography Standard) and parent age group using Australian Bureau of Statistics (ABS) Census 2021 data. The design weight, the ratio of the population proportion to the sample proportion, was used. Where relevant, we collapsed the number of categories to avoid the distortion of the data due to small cell sizes. Parent age was collapsed into two categories for this reason.

Demographic characteristics were described using frequencies, unweighted percentages and weighted percentages. Previous influenza vaccination behaviour, future intention and the prevalence of vaccination barriers were described using weighted data.

For the primary analysis, a generalised linear model with a binomial distribution (binomial regression model) estimated the difference in the prevalence of each vaccination barrier between parents who were intending to and those who were unsure or not intending to have their youngest child vaccinated against influenza in 2024. The prevalence difference (PD) and 95% confidence interval (CI) were presented for each vaccination barrier.

Secondary analyses compared vaccination barriers (limited to those with the largest prevalence differences, i.e., where the lower bound of 95% CI was ~10% or greater between the intending to vs. unsure/not intending to vaccinate groups) by key demographic variables: parent’s regionality (urban vs. rural areas), parent’s experience of financial stress (experiencing vs. not experiencing financial stress) and number of children in the family (one child vs. multiple children). A binomial regression model estimated the prevalence difference in each vaccination barrier between these demographic groupings.

This study was reported in accordance with the STROBE (Strengthening the Reporting of Observational Studies in Epidemiology) guidelines for cross-sectional studies [27].

### 2.10. Ethical Considerations

The Sydney Children’s Hospital Human Research Ethics Committee (2023/ETH02177) gave ethical approval for this study. Participants gave informed consent by reading a participant information and consent form and indicating their consent digitally.

## 3. Results

### 3.1. Participant Characteristics

Demographic characteristics of the participants are shown in Table 1. For previous influenza vaccination behaviour, 69.2% of parents reported that their youngest child had received an influenza vaccine prior to the 2024 influenza season (in 2023 or earlier). For future intentions, 75.3% said they intended to give their youngest child an influenza vaccine in the May–September 2024 influenza season, while 24.8% were either unsure (10.4%) or not intending to vaccinate (14.4%).

### 3.2. Prevalence of Influenza Vaccination Barriers

**Figure 1:**
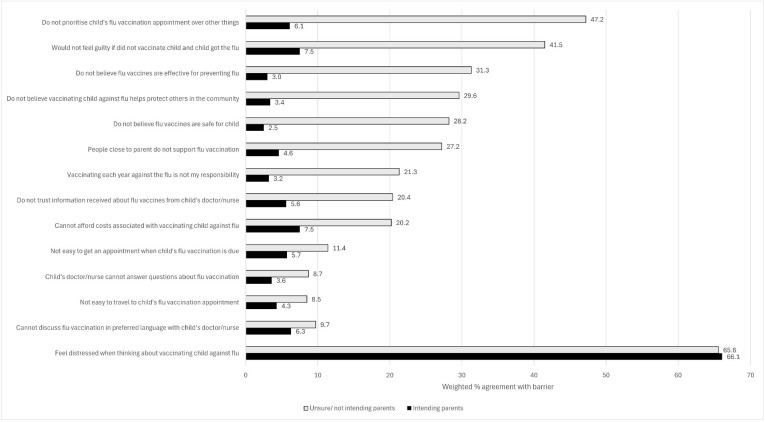
Prevalence of barriers to childhood influenza vaccination by parental intention to vaccinate.

The most common barrier to influenza vaccination was related to the parent’s distress, with 65.6% of unsure/not intending to vaccinate parents and 66.1% of intending to vaccinate parents reporting feeling distressed when thinking about vaccinating their child against influenza (Figure 1).

Among unsure/not intending parents, the second most reported barrier to influenza vaccination was not prioritising their child’s influenza vaccination appointment over other things (47.2%). The least reported barrier was not finding it easy to travel to their child’s vaccination appointment (8.5%).

The proportion of intending parents reporting any barrier other than distress was notably lower in comparison to unsure/not intending parents. Among intending parents, the most reported barriers (after distress) were not being able to afford costs associated with vaccinating their child against influenza (7.5%) and not feeling guilty if their unvaccinated child got influenza (7.5%). The least reported barrier in this group was not believing influenza vaccines were safe for their child (2.5%).

See Figure S1 in the Supplemental Materials for the prevalence of agreement with barriers by intention group, showing all response categories.

### 3.3. Comparing Influenza Vaccination Barriers by Parent’s Intention to Vaccinate

**Figure 2:**
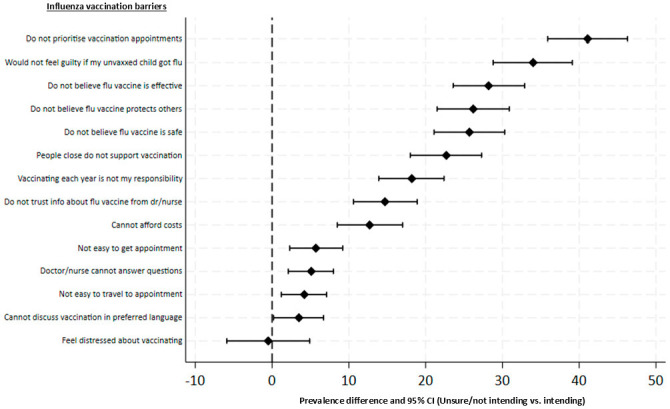
Differences in prevalence of influenza vaccination barriers comparing unsure/not intending parents with intending parents.

Prevalence differences and 95% CIs for all barriers are presented in Table 2 and Figure 2. Around two-third of parents reported feeling distressed when thinking about vaccinating their child against influenza, irrespective of whether they intended (66.1%) or did not intend (65.6%) to have their child vaccinated in 2024. The barrier with the largest difference between intending and not intending/unsure parents was not prioritising their child’s influenza vaccination appointment over other things (PD 41.1%; 95% CI 35.9% to 46.3%). Other barriers with large prevalence differences included not feeling guilty if their unvaccinated child got influenza; not believing that influenza vaccines were effective for preventing influenza; not believing that vaccinating their child against influenza protected others in the community; and not believing influenza vaccines were safe for their child. The barrier with the smallest difference was being unable to discuss vaccination in the parent’s preferred language (PD 3.5%; 95% CI 0.2% to 6.7%).

### 3.4. Comparing Influenza Vaccination Barriers by Key Demographic Variables

Fewer parents living in rural areas reported not prioritising influenza vaccination over other things, compared to parents living in urban areas (PD −6.1%; 95% CI −10.7% to −1.5%).

The barrier with the largest difference between parents experiencing financial stress and those who were not was being unable to afford the costs associated with vaccinating (PD 7.4%; 95% CI 4.0% to 10.8%). Parents experiencing financial stress were also more likely to report not believing that influenza vaccines were safe for their child (PD 4.3%; 95% CI 1.3% to 7.4%).

The percentage of parents who agreed with each barrier (except two) increased with the number of children in the family. The barrier with the largest difference between parents with one and multiple children was not prioritising their child’s influenza vaccination appointment over other things. The prevalence difference for parents with two children was 10.7% (95% CI 6.7% to 14.6%) and for parents with three or more children was 17.0% (95% CI 10.7% to 23.2%) compared to parents with one child. See Table S1 in Supplemental Materials for full results.

## 4. Discussion

This study found that parents with a range of intentions experience barriers to vaccinating their child against influenza. The most common barrier in both unsure/not intending and intending parents was feeling distressed when thinking about vaccinating their child. Studies in other countries have also identified parental distress related to vaccination, with some finding an association with negative vaccination attitudes or behaviour [28,29,30]. In this study, however, parental distress was not found to deter parents from intending to vaccinate, with a similar proportion of unsure/not intending and intending parents reporting distress.

The barriers with the largest differences between intending and unsure or not intending parents are more likely to influence vaccination intention and are therefore important targets for intervention efforts aimed at increasing uptake. Among parents of children <5 years in Australia, the barrier with the largest difference was not prioritising influenza vaccination over other things.

Not prioritising can be a result of choice, i.e., the parent chooses not to prioritise influenza vaccination. Previous Australian research has found that some parents choose not to prioritise influenza vaccination because they view it as less important than routine childhood vaccines [22,23]; similar results have been found in other countries such as the United Kingdom and United States [31,32]. Other reasons parents may choose not to prioritise influenza vaccination are identified by this study as significant barriers to intention, for example, they may perceive influenza to be low risk and their children to not be susceptible, leading to less anticipated guilt. This is supported by evidence from other studies, where parents who perceived influenza to be low risk were less likely to vaccinate their child against influenza [33]. Another reason for low prioritisation, also identified as a barrier in this study, may be parents perceiving the influenza vaccination to be ineffective. Other studies have also reported parents expressing reluctance to vaccinate their children against influenza because of perceived vaccine ineffectiveness, saying they know people who “got the flu” despite being vaccinated [22,34]. Research has also found that parents perceive influenza vaccines to be less effective than routine childhood vaccines [32].

Alternatively, not prioritising can be a result of practical difficulties, i.e., the parent is not able to prioritise influenza vaccination because of practical or access issues. In this study, the differences between intending and unsure or not intending parents reporting practical barriers were smaller than those for motivational or acceptance barriers. However, this may be because this study used intention to vaccinate as the outcome of interest. Using actual vaccination behaviour as the outcome may better capture the impact of practical barriers. This notion is supported by research examining vaccination barriers beyond those related to intention, which has found that parents are not able to prioritise influenza vaccination due to practical difficulties: parents report inconveniences and practical challenges, such as requirements for annual influenza vaccination and juggling the multiple steps required to complete vaccination, including booking appointments, managing competing priorities to get their child to the clinic and taking time off work [22]. Our study did find that parents with multiple children were less likely to prioritise influenza vaccination and reported an increased number of practical barriers including cost, while parents experiencing financial stress also reported cost as a barrier. In other studies, parents and providers have also reported cost as a barrier to childhood influenza vaccination, describing how paying to see the child’s doctor and losing work hours can quickly add up, especially for families with multiple children [22]. This suggests that parents’ ability to prioritise influenza vaccination, as well as manage some of the associated barriers like cost, may be influenced by practical, equity-related factors such as cost-of-living pressures and the demands of caring for multiple children.

Our analysis revealed that parents who were unsure or not intending to vaccinate their child against influenza were more likely to believe influenza vaccination posed a safety risk for their child. Our findings resonate with previous research, which has found that parents are concerned about influenza vaccine safety [22,23,34]. Enduring misperceptions, especially that influenza vaccines cause influenza [35], may help explain these findings. Previous vaccine safety scares, such as the 2010 event where an unexpected number of children in Western Australia experienced severe febrile reactions after vaccination with a specific brand of influenza vaccine [36], may also play a continuing role in shaping parents’ perceptions of the risks associated with influenza vaccination [37]. Our analysis also found that those parents experiencing financial stress were more likely to report not believing influenza vaccines were safe for their child; evidence that lower income is associated with less trust in the healthcare systems that deliver vaccination may help explain this finding [38].

Finally, our analysis found that parents who were unsure or not intending to vaccinate their child against influenza were more likely to report a lack of trust in vaccination information from healthcare providers. Research on factors influencing routine childhood vaccinations has also found a similar lack of trust influencing vaccination decisions [39,40]. Some researchers have suggested that lack of trust in science or the health system drives vaccine hesitancy, which may be what our study is identifying [41]. Encouragingly, however, recent research finds global trust in experts (in this case scientists) to be moderately high, challenging the notion of widespread distrust in experts more broadly [42].

### 4.1. Strengths and Limitations

A strength of this study is its national sample, which closely reflects the Australian parent population in terms of gender and location. While we had fewer younger parents aged 18–29 years, the proportion of parents aged 30–45 years was very similar to the Australian population. To enhance representativeness, the sample was randomly drawn from a large, established panel using stratified sampling by state and territory. Although the panel itself is not a probability sample of the general population, and therefore some caution is warranted in generalising results to all Australian parents, the panel is constructed to approximate the national population and weighting further improved the representativeness of the sample. These approaches enhanced the external validity of the findings. Based on a comparison with the population data provided by the ABS, no large discrepancies were identified. There was a potential for selection bias and non-generalisability of findings due to using an online panel for recruitment, as such panels may not adequately represent certain populations, such as culturally and linguistically diverse people. A further limitation was the exclusion of participants who could not read/understand English, which may have influenced our data on barriers, in particular related to discussing vaccination in a parent’s preferred language. A higher proportion of participants reported previously vaccinating their child against influenza than was expected given annual coverage rates. It was not possible to calculate an accurate response rate or compare respondents and nonrespondents due to the way in which the panel recruited participants to the study. We did, however, use measures to minimise selection bias. For example, the recruitment text and participant information sheet and consent form provided only brief, neutral information about the study topic. This approach could help reduce the likelihood of participants self-selecting for the study based on strong interest in or views on the topic, which could distort the sample and limit generalisability.

A further strength is the VBAT survey tool, which ensures the robust measurement of parental barriers to vaccination. Also, this study established a baseline and method for collecting national data on vaccination barriers, which will enable tracking and comparisons over time. Not only are data on barriers an important and long-overdue addition to the suite of vaccination-related data (e.g., coverage and disease surveillance) in Australia, they will also prove critical during times when vaccination becomes an even more pressing societal issue, such as during future pandemics. Although the VBAT is a validated tool for assessing barriers to routine childhood vaccination, the adapted version used in this study has not been formally validated for influenza vaccination, which may affect the measurement of barriers in this context. However, the underlying constructs of the VBAT are conceptually relevant to influenza vaccination, and care was taken to adapt only the wording while preserving the original intent of each item. Our item measuring the barrier of prioritising vaccination might be interpreted by participants as either a choice or a practical issue. In future applications, this item should be amended to specify that it relates to choice (the original objective).

Finally, we were only able to measure vaccination intention as an outcome due to challenges accessing uptake data. This may have led to a stronger representation of factors more closely related to acceptance (proximal to intentions). Intention, however, remains a useful outcome measure given the timing of the study (right before the start of the influenza season). In future iterations of this study, once data linkage and privacy issues are resolved, we intend to use de-identified uptake data as our outcome measure. Further, the next iteration of this study will collect data immediately after the influenza season has ended when parents’ recollection of uptake is fresh. Self-report is an accepted means of measuring uptake and is used in many countries without accurate immunisation registers.

### 4.2. Implications

The implication of parents not prioritising vaccinating their child against influenza, having concerns about vaccine effectiveness and safety and not trusting information from healthcare workers is, ultimately, lower influenza vaccination rates. This in turn leads to increased disease in the community and more children experiencing complications and hospitalisation. Understanding factors hindering influenza vaccination can inform strategies to encourage uptake, which is especially pertinent in the face of suboptimal and declining coverage.

These findings suggest that programmes should focus on helping parents prioritise vaccination by emphasising the risk of severe disease even in healthy children and that influenza vaccination is equally as important as other childhood vaccinations. Public health campaigns should aim to improve parental beliefs about influenza vaccination by fact-checking misperceptions and providing up-to-date information about vaccine safety and effectiveness.

It is possible that more accessible influenza vaccination services could help parents experiencing practical difficulties prioritise vaccination. Offering vaccination in pharmacies, pop-up or walk-in vaccination clinics and where young children congregate, i.e., in childcare and shopping centres, could make influenza vaccination easier to access for time- and resource-poor parents. Healthcare workers should be supported to recommend and offer influenza vaccination opportunistically, not just during dedicated vaccination appointments.

### 4.3. Future Research

We recommend conducting serial surveys to monitor trends in parental barriers to childhood influenza vaccination to inform strategies over time. Future research could explore associations between uptake and pre-existing health conditions. Efforts should be made to capture the views of participants who do not read or understand English. Qualitative research could examine how parents’ trust in influenza vaccination information from their child’s nurse or doctor continues to evolve in the post-pandemic context. Validating the adapted VBAT would ensure it reliably measures parental influenza vaccination barriers.

## 5. Conclusions

Among parents of children < 5 years in Australia, barriers with large differences between intending and not intending or unsure parents included not prioritising influenza vaccination, having concerns about vaccine effectiveness and safety and not trusting information from healthcare workers. Parents should be encouraged and supported to prioritise influenza vaccination alongside other routine childhood vaccines via campaigns that emphasise disease risk and the importance, safety and effectiveness of influenza vaccination, and by optimising access to vaccination, with consideration given to costs. Serial surveys should be conducted to monitor trends in parental barriers to childhood influenza vaccination to inform strategies over time.

## Figures and Tables

**Table 1:** Participant characteristics.

	Unweighted		Weighted	ABS Data on Australian Parents of Children <5
	*n*	(%)	(%)	%
**N**	2000	-	-	-
**Gender**				
Woman	1091	(54.5%)	(52.3%)	53.8%
Man	901	(45.1%)	(47.4%)	46.3%
Non-binary	3	(0.1%)	(0.1%)	no data
Prefer not to say	5	(0.2%)	(0.2%)	-
**Age (years)**				
18 to 24	32	(1.6%)	(1.3%)	3.4%
25 to 29	153	(7.6%)	(6.8%)	12.9%
30 to 34	742	(37.1%)	(38.1%)	29.9%
35 to 39	743	(37.1%)	(37.6%)	32.4%
40 to 44	291	(14.5%)	(14.3%)	15.1%
45 to 49	29	(1.5%)	(1.4%)	4.1%
50 to 54	7	(0.4%)	(0.3%)	1.2%
55 to 59	3	(0.1%)	(0.1%)	0.5%
**State or territory**				
New South Wales	612	(30.6%)	(32.8%)	32.0%
Victoria	517	(25.9%)	(27.3%)	26.6%
Queensland	404	(20.2%)	(19.7%)	19.3%
Western Australia	217	(10.8%)	(11.0%)	10.8%
South Australia	154	(7.7%)	(6.6%)	6.45%
Tasmania	47	(2.4%)	(0.4%)	1.9%
Australian Capital Territory	36	(1.8%)	(2.0%)	1.9%
Northern Territory	13	(0.7%)	(0.3%)	1.0%
**Regionality**				
Metropolitan	1458	(72.9%)	(92.4%)	
Regional	348	(17.4%)	(4.8%)	
Rural	159	(8.0%)	(2.3%)	
Remote	35	(1.8%)	(0.5%)	
**Aboriginal and Torres Strait Islander status**				
Aboriginal	57	(2.9%)	(2.3%)	
Torres Strait Islander	11	(0.5%)	(0.4%)	
Both	23	(1.1%)	(0.8%)	
Neither	1909	(95.5%)	(96.5%)	
**Education**				
Less than high school	13	(0.7%)	(0.6%)	
High school or equivalent	324	(16.2%)	(14.3%)	
Trade cert/apprenticeship	442	(22.1%)	(20.6%)	
Bachelor’s degree	879	(44.0%)	(46.3%)	
Graduate degree	342	(17.1%)	(18.2%)	
**Language used at home**				
English only	1739	(87.0%)	(84.8%)	
Other language	261	(13.1%)	(15.2%)	
**Number of children**				
1	1010	(50.5%)	(53.3%)	
2	697	(34.8%)	(34.0%)	
3+	293	(14.6%)	(12.8%)	
**Single parent or carer**				
Yes	1072	(53.6%)	(55.8%)	
No	928	(46.4%)	(44.2%)	
**Financial stress**				
Yes	726	(36.3%)	(34.4%)	
No	1274	(63.7%)	(65.6%)	
**Youngest child’s age**				
<1 year	192	(9.6%)	(9.1%)	
1 to <2 years	359	(17.9%)	(17.1%)	
2 to <3 years	507	(25.4%)	(25.3%)	
3 to <4 years	519	(25.9%)	(25.9%)	
4 to <5 years	423	(21.1%)	(22.6%)	

**Table 2:** Differences in prevalence of influenza vaccination barriers between unsure/not intending parents and intending parents of children aged <5 years.

Influenza Vaccination Barrier	PD	95% CI Lower	Upper
Do not prioritise child’s flu vaccination appointment over other things	41.1%	35.9%	46.3%
Would not feel guilty if they did not vaccinate child and child got the flu	34.0%	28.8%	39.1%
Do not believe flu vaccines are effective for preventing flu	28.2%	23.6%	32.9%
Do not believe vaccinating child against flu helps protect others in the community	26.2%	21.5%	30.9%
Do not believe flu vaccines are safe for child	25.7%	21.1%	30.3%
People close to parent do not support flu vaccination	22.7%	18.0%	27.3%
Vaccinating each year against the flu is not parent’s responsibility	18.2%	13.9%	22.4%
Do not trust information received about flu vaccines from child’s doctor/nurse	14.7%	10.6%	18.9%
Cannot afford costs associated with vaccinating child against flu	12.7%	8.5%	17.0%
Not easy to get an appointment when child’s flu vaccination is due	5.7%	2.3%	9.2%
Child’s doctor/nurse cannot answer questions about flu vaccination	5.1%	2.1%	8.0%
Not easy to travel to child’s flu vaccination appointment	4.2%	1.2%	7.1%
Cannot discuss flu vaccination in preferred language with child’s doctor/nurse	3.5%	0.2%	6.7%
Feel distressed when thinking about vaccinating child against flu	−0.5%	−5.9%	4.9%

## Data Availability

The data presented in this study are not available due to privacy and ethical restrictions.

## Supplementary Materials

The following supporting information can be downloaded at: https://www.mdpi.com/article/10.3390/vaccines13050540/s1: STROBE checklist; Survey instrument; Figure S1. Distribution of responses to childhood influenza vaccination barriers by parental intention to vaccinate; Table S1. Difference in percentage of parents of children aged <5 years reporting influenza vaccination barriers, by key demographic variables.
