# Supplementary material for: Childhood Influenza Vaccination Is Not a Priority for Parents: A National, Cross-Sectional Survey of Barriers to Childhood Influenza Vaccination in Australia
Source: Vaccines (Basel). 2025 May 19;13(5):540. doi: 10.3390/vaccines13050540 (PMC12115364; doi:10.3390/vaccines13050540)
Supplement: Supplementary file 1 [file vaccines-13-00540-s001.zip › vaccines-3597334-supplementary.pdf]

Supplemental materials

Figure S1. Distribution of responses to childhood influenza vaccination barriers by parental intention to vaccinate

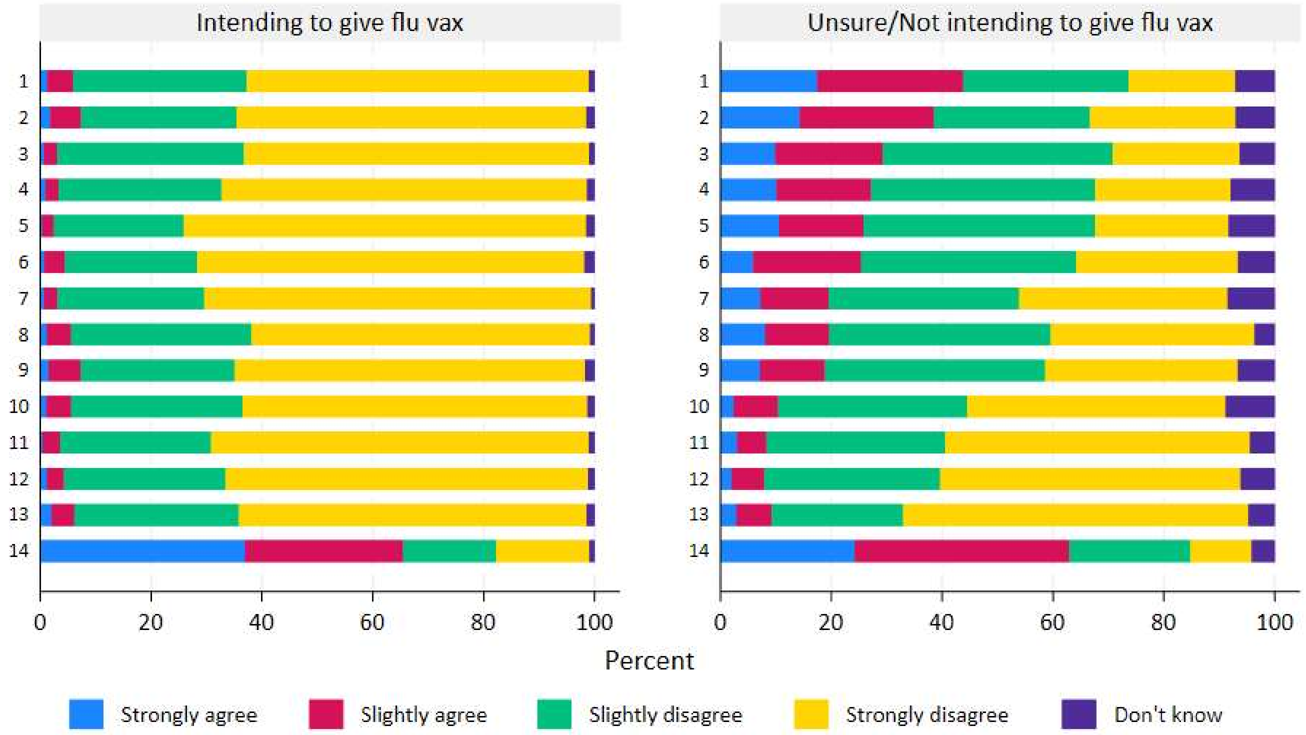

Key – Influenza vaccination barriers

1	Do not prioritise child's vaccination appointments over other things	8	Do not trust info received about flu vaccines from child's dr/nurse
2	Would not feel guilty if did not vaccinate and child got flu	9	Cannot afford costs associated with vaccinating child against flu
3	Do not believe flu vaccines are effective for preventing flu	10	Not easy to get appointment when child's flu vaccine is due
4	Do not believe flu vaccine protects others in the community	11	Doctor/nurse cannot answer questions about flu vaccination
5	Do not believe flu vaccines are safe for child	12	Not easy to travel to child's flu vaccination appointment
6	People close to parent do not support flu vaccination	13	Cannot discuss vaccination in preferred language with child's dr/nurse
7	Vaccinating each year against the flu is not my responsibility	14	Feel distressed when thinking about vaccinating child against flu

Table S1. Difference in percentage of parents of children aged < 5 years reporting influenza vaccination barriers, by key demographic variables

	Living in urban* v. rural area			Not experiencing* v. experiencing financial stress			Having 1* v. 2 children			Having 1* v. 3 or more children		
		95% CI			95% CI			95%CI			95%CI	
**Influenza vaccination barrier**	PD	Lower	Upper	PD	Lower	Upper	PD	Lower	Upper	PD	Lower	Upper
Do not prioritise child's flu vaccination appointment over other things	-6.1	-10.7	-1.5	-0.5	-4.2	3.2	10.7	6.7	14.6	17.0	10.7	23.2
Would not feel guilty if did not vaccinate child and child got the flu	-2.1	-7.4	3.1	0.2	-3.5	3.8	9.2	5.3	13.1	15.4	9.3	21.5
Do not believe flu vaccines are effective for preventing flu	0.3	-4.1	4.7	4.0	0.8	7.2	4.9	1.7	8.1	6.4	1.7	11.2
Do not believe vaccinating child against flu helps protect others	-1.1	-5.4	3.3	-0.3	-3.3	2.6	5.8	2.6	9.1	7.5	2.7	12.3
Do not believe flu vaccines are safe for child	-3.0	-6.4	0.4	4.3	1.3	7.4	4.5	1.4	7.6	5.5	1.1	10.0
People close to parent do not support flu vaccination	-1.6	-5.8	2.6	0.4	-2.8	3.5	5.3	1.9	8.7	3.0	-1.4	7.5
Vaccinating each year against the flu is not parent's responsibility	-2.6	-5.8	0.7	0.5	-2.2	3.2	4.8	1.8	7.7	4.9	0.8	9.1
Do not trust information received about flu vaccines from doctor/nurse	-1.0	-5.2	3.1	2.4	-0.6	5.4	3.5	0.3	6.6	4.6	0.0	9.2
Cannot afford costs associated with vaccinating child against flu	-3.1	-7.0	0.8	7.4	4.0	10.8	5.7	2.3	9.0	8.4	3.2	13.6

*reference group

Survey instrument

First, we'd like to ask you a few questions about yourself.
What is your age?<br><br>[open-ended]
How many children do you have?<br><br>1, 2, 3, 4, 5, 6+, I do not have any children*
How many of your children are under five years old?<br><br>0*, 1, 2, 3, 4, 5, 6+
Thinking about your youngest child, in what month and year were they born??<br><br>[drop down month] + [drop down year]
What is your post code?<br><br>[open ended]<br>I don't live in Australia*
What is your gender?<br><br>Woman<br>Man<br>Non-binary<br>Prefer not to say
Are you of Aboriginal or Torres Strait Islander origin?<br><br>No<br>Yes, Aboriginal<br>Yes, Torres Strait Islander<br>Yes, both Aboriginal and Torres Strait Islander
What is the highest level of education you have completed?<br><br>Less than high school<br>High school or equivalent<br>Trade certificate/apprenticeship<br>Bachelor's degree<br>Graduate degree (undertaken after a bachelor's degree, e.g. Master's degree, doctorate)
Do you use a language other than English at home? If you speak more than 1 language, please select the language you speak most often at home.<br><br>No, English only

Yes, Mandarin<br>Yes, Arabic<br>Yes, Cantonese<br>Yes, Vietnamese<br>Yes, Italian<br>Yes, Greek<br>Yes, other language (please specify) [free text]
Are you a <u>single</u> parent or carer to one or more children under the age of five?<br><br>Yes<br>No
Since January 2023 did any of the following happen to you because of a shortage of money?<br><br>Could not pay electricity, gas or telephone bills on time<br><br>Could not pay the mortgage or rent on time<br><br>Pawned or sold something<br><br>Went without meals<br><br>Was unable to heat home<br><br>Asked for financial help from friends or family<br><br>Asked for help from welfare/community organisations<br><br>None of these
Now we will ask you some questions about the seasonal influenza (flu) vaccine. Please answer about your youngest child.
Did your child have the flu vaccine any time before 2024 (e.g. in 2023 or before)?<br><br>Yes<br>No<br>Not sure
Do you want your child to get an influenza (flu) vaccine in the upcoming flu season (May-September 2024)?<br><br>Yes<br>No<br>Not sure
Please select only one answer to each of the statements below.<br><br>1. It is easy to get an appointment for my child's flu vaccination<br>2. It is easy for me to travel to my child's flu vaccination appointment<br>3. I believe the flu vaccine is effective for preventing the flu<br>4. I believe vaccinating my child against the flu helps protect others in the community

5. I believe the flu vaccine is safe for my child
6. I would feel guilty if I did not give my child the flu vaccine and they got the flu
7. I feel distressed when I think about giving my child the flu vaccine
8. I can afford any costs associated with giving my child the flu vaccine
9. I can discuss flu vaccination in my preferred language with my child's doctor or nurse
10. It is my responsibility to make sure my child gets the flu vaccine each year
11. I prioritise my child's flu vaccination appointment over other things
12. People close to me support flu vaccination
13. My child's doctor or nurse can answer my questions about flu vaccination
14. I trust the information I receive about the flu vaccine from my child's doctor or nurse

Strongly disagree
Slightly disagree
Slightly agree
Strongly agree
Don’t know/can’t say

STROBE Statement—Checklist of items that should be included in reports of *cross-sectional studies*

	Item No	Recommendation	Page No^
**Title and abstract**	1	(*a*) Indicate the study's design with a commonly used term in the title or the abstract	1
		(*b*) Provide in the abstract an informative and balanced summary of what was done and what was found	3
**Introduction**			
Background/rationale	2	Explain the scientific background and rationale for the investigation being reported	5-7
Objectives	3	State specific objectives, including any prespecified hypotheses	7
**Methods**			
Study design	4	Present key elements of study design early in the paper	7
Setting	5	Describe the setting, locations, and relevant dates, including periods of recruitment, exposure, follow-up, and data collection	7
Participants	6	(*a*) Give the eligibility criteria, and the sources and methods of selection of participants	8
Variables	7	Clearly define all outcomes, exposures, predictors, potential confounders, and effect modifiers. Give diagnostic criteria, if applicable	8-9
Data sources/ measurement	8	For each variable of interest, give sources of data and details of methods of assessment (measurement). Describe comparability of assessment methods if there is more than one group	9
Bias	9	Describe any efforts to address potential sources of bias	10
Study size	10	Explain how the study size was arrived at	10
Quantitative variables	11	Explain how quantitative variables were handled in the analyses. If applicable, describe which groupings were chosen and why	10
Statistical methods	12	(*a*) Describe all statistical methods, including those used to control for confounding	11
		(*b*) Describe any methods used to examine subgroups and interactions	NA
		(*c*) Explain how missing data were addressed	10-11
		(*d*) If applicable, describe analytical methods taking account of sampling strategy	10-11
		(*e*) Describe any sensitivity analyses	NA

Results

Participants	13*	(a) Report numbers of individuals at each stage of study—eg numbers potentially eligible, examined for eligibility, confirmed eligible, included in the study, completing follow-up, and analysed	12-13
		(b) Give reasons for non-participation at each stage	NA
		(c) Consider use of a flow diagram	NA
Descriptive data	14*	(a) Give characteristics of study participants (eg demographic, clinical, social) and information on exposures and potential confounders	12-13
		(b) Indicate number of participants with missing data for each variable of interest	NA
Outcome data	15*	Report numbers of outcome events or summary measures	14-16
Main results	16	(*a*) Give unadjusted estimates and, if applicable, confounder-adjusted estimates and their precision (eg, 95% confidence interval). Make clear which confounders were adjusted for and why they were included	18
		(*b*) Report category boundaries when continuous variables were categorized	NA
		(*c*) If relevant, consider translating estimates of relative risk into absolute risk for a meaningful time period	NA
Other analyses	17	Report other analyses done—eg analyses of subgroups and interactions, and sensitivity analyses	20
**Discussion**			
Key results	18	Summarise key results with reference to study objectives	20-22
Limitations	19	Discuss limitations of the study, taking into account sources of potential bias or imprecision. Discuss both direction and magnitude of any potential bias	22-23
Interpretation	20	Give a cautious overall interpretation of results considering objectives, limitations, multiplicity of analyses, results from similar studies, and other relevant evidence	20-22
Generalisability	21	Discuss the generalisability (external validity) of the study results	22-23
**Other information**			
Funding	22	Give the source of funding and the role of the funders for the present study and, if applicable, for the original study on which the present article is based	27

^Note: Page numbers refer to the version submitted to the journal. Due to formatting differences, they may not correspond to the reviewer or final version.
